# Additive Manufacturing Techniques for Acoustic Hologram Lens Microfabrication

**DOI:** 10.3390/mi16101119

**Published:** 2025-09-29

**Authors:** Jinwook Kim, Hoseok Kang, Seok Choi, Doyoon Kim

**Affiliations:** School of Mechanical Engineering, Kyungpook National University, Daegu 41566, Republic of Korea

**Keywords:** acoustic holograms, meta structure, kinoform, phase delay, additive manufacturing

## Abstract

Acoustic hologram lenses enable the precise shaping of sound fields using plane wave inputs, with applications in biomedical imaging, targeted therapy, and acoustic manipulation. Recent advances in additive microfabrication technologies have greatly improved the design and fabrication of these lenses supporting materialization of complex phase profiles, miniaturization, and rapid prototyping. This review summarizes key progress in fabrication methods including stereolithography, material jetting, and fused deposition modeling that have expanded the versatility and performance of acoustic hologram lenses. We examine the fabrication techniques, operating frequencies, printing resolutions, and acoustic properties reported in the literature. This review provides an organized overview of the current state of acoustic hologram lens fabrication and highlights critical challenges and future opportunities for advancing research and practical applications.

## 1. Introduction

Acoustic holograms have emerged as a useful ultrasonic energy delivery tool for the precise manipulation of ultrasound fields, owing to the capability of reconstructing micro-resolution, multi-focal pressure distributions in an acoustic medium [1,2]. This pressure distribution shaping technique has stimulated intensive research activity in recent years and has enabled a wide spectrum of engineering applications [3], including transcranial ultrasound therapies [4], cavitation control [5,6], particle manipulation [7,8,9], tissue ablation [10], and bio-sample processing [11].

To realize acoustic holograms, most studies have relied on additive manufacturing (3D printing), which has become the dominant prototyping approach [3,12]. Among various techniques, stereolithography (SLA) has gained particular traction due to its ability to rapidly fabricate holographic lenses with high spatial resolution (<100 µm) while suppressing unintended porosity [13]. SLA is now regarded as a mature and efficient printing technology, offering both precision and cost-effectiveness. Using this method, many studies have demonstrated accurate pressure amplitude shaping within the excitation frequency range of 1–2 MHz [3,14]. Similar photopolymers have also been employed in material jetting, or PolyJet modeling, for acoustic hologram lens fabrication, extending the frequency range up to 3 MHz [15]. Both SLA and material jetting rely on photopolymer curing, enabling fine spatial resolution (<100 µm) in fabricated hologram lenses [16]. In contrast, fused deposition modeling (FDM) has been applied primarily in multifocal transcranial ultrasound treatments at 1–2 MHz [4]. Although simpler and more cost-efficient than SLA or material jetting, FDM typically offers lower resolution (hundreds of microns) than photopolymer prototypes [17].

Despite the dominance of additive manufacturing in acoustic hologram fabrication, clear technical limitations remain. The performance of fabricated lenses through SLA, material jetting, or FDM is fundamentally constrained by the acoustic properties of commercial resins and filaments. With densities of 1000–1300 kg/m^3^ and sound speeds of 1700–2300 m/s, their corresponding acoustic impedances fall within 1.7–3 MRayl. This limited impedance range contrasts with the broader material options available for conventional ultrasonic transducer assemblies [18,19], leading to significant impedance mismatch and reduced transmission efficiency, and these have been critical barriers to high-pressure output performance implementations. To overcome these challenges, recent studies have explored hybrid fabrication strategies that combine 3D printing with conventional composite molding methods, aiming to improve material versatility, acoustic efficiency, and device functionality [20]. These innovations continue to expand the design space and application potential of acoustic holograms [11,21].

Acoustic holograms have emerged as a significant branch of the broader research field of acoustic metamaterials and metasurfaces [22,23,24]. Despite rapid progress in the past decade, a systematic review that critically examines fabrication methods, advantages, limitations, and prototyping outcomes remains lacking. This review addresses that gap by providing a comprehensive survey of acoustic hologram microfabrication technologies, emphasizing their technical principles, trade-offs, and acoustic suitability for diverse application domains. By consolidating these insights, it aims to serve as a practical guideline for both hologram designers and engineers developing customized multifocal ultrasonic systems.

## 2. Background for Acoustic Holograms and Literature Survey

### 2.1. Design of Acoustic Holograms by Iterative Angular Spectrum Approach

Acoustic holograms encode a specific phase distribution on a two-dimensional plane to generate a predefined multi-focal pressure amplitude distribution at a target plane, typically located within the Fresnel zone [1,25]. The design process generally involves simulating a two-dimensional planar wavefield, followed by layered propagation analysis, which aligns well with the angular spectrum method [26]. Among the various acoustic theories and algorithms for designing acoustic holograms, such as diffraction [27,28,29,30], the most widely used computational technique is the Iterative Angular Spectrum Approach (IASA), which performs repeated forward and backward propagations, often more than 50 iterations, to accurately reconstruct the target pressure field as demonstrated by our previous studies [20,21]. The acoustic pressure field is expressed as Equation (1) where $\hat{p}\left(x,y,z\right)$ is the harmonic pressure amplitude and $e^{j\Delta \phi \left(x,y,z\right)}$ is the harmonic phase function [31].

(1) $$p\left(x,y,z\right)=\hat{p}\left(x,y,z\right)\cdot e^{j\phi \left(x,y,z\right)}$$

The phase function at the hologram plane (*z* = 0) is pixelized to yield the hologram map corresponding to the desired amplitude distribution at the target plane (*z* = *z*_*t*_). Once the target map accuracy is confirmed, the final phase map is converted into a lens thickness profile using the following relations, where *k* is the wavenumber, *t* is the lens thickness, and the subscripts *m* and *h* denote the propagation medium (typically water) and the hologram lens material, respectively [1].

(2) $$\Delta \phi \left(x,y\right)=\left(k_{m}-k_{h}\right)\Delta t\left(x,y\right)$$

(3) $$t\left(x,y\right)=t_{0}-\Delta t\left(x,y\right)$$

An important factor in hologram design is the transmission coefficient, which governs acoustic energy transfer efficiency from the source to the target plane. After determining pixel thicknesses from the phase relation, the transmission coefficient at each pixel is calculated as Equation (4), where *Z* represents acoustic impedance, with subscripts *t*, *h*, and mmm referring to the source (e.g., piezoelectric transducer), the hologram lens, and the medium, respectively, Thus, the pressure amplitude at the hologram plane (*z* = 0) can be expressed as Equation (5) [21,31].

(4) $$T\left(x,y\right)=\frac{4Z_{t}Z_{h}^{2}Z_{m}}{Z_{h}^{2}{\left(Z_{t}+Z_{m}\right)}^{2}\cos^{2}\left(k_{h}t\left(x,y\right)\right)+{\left(Z_{h}^{2}+Z_{t}Z_{m}\right)}^{2}\sin^{2}\left(k_{h}t\left(x,y\right)\right)}$$

(5) $$\hat{p}\left(x,y,0\right)=T\left(x,y\right)\;{\hat{p}}_{0}\left(x,y\right)$$

**Figure 1:**
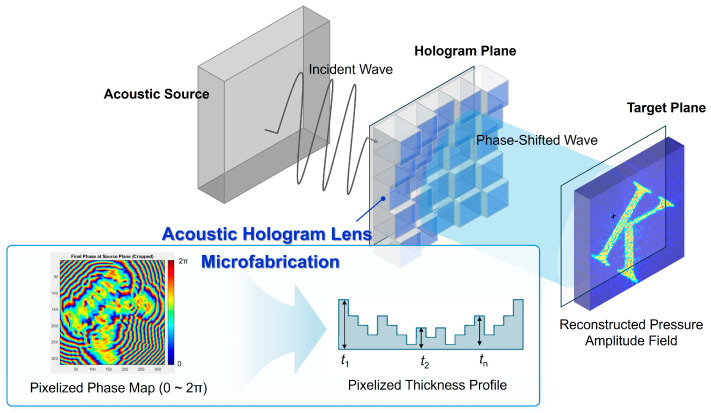
Schematic of an acoustic hologram lens and microfabrication of lens structure.

Recently, various machine learning (ML) techniques have been applied to design two-dimensional phase maps for acoustic hologram lenses. Compared to IASA or other theoretical or numerical simulation methods, ML-assisted design offers significantly (more than 100 times) faster computation and optimization [32,33,34,35,36,37]. Regardless of whether the design follows a theoretical simulation approach or an ML-assisted workflow, the resulting acoustic hologram lens is translated into a microscale thickness distribution, converted into a 3D model (STL file), and prepared as a layered structure (g-code) for additive manufacturing [5]. A schematic overview of the acoustic hologram lens is presented in Figure 1.

### 2.2. Lituerature Survey Approach

**Figure 2:**
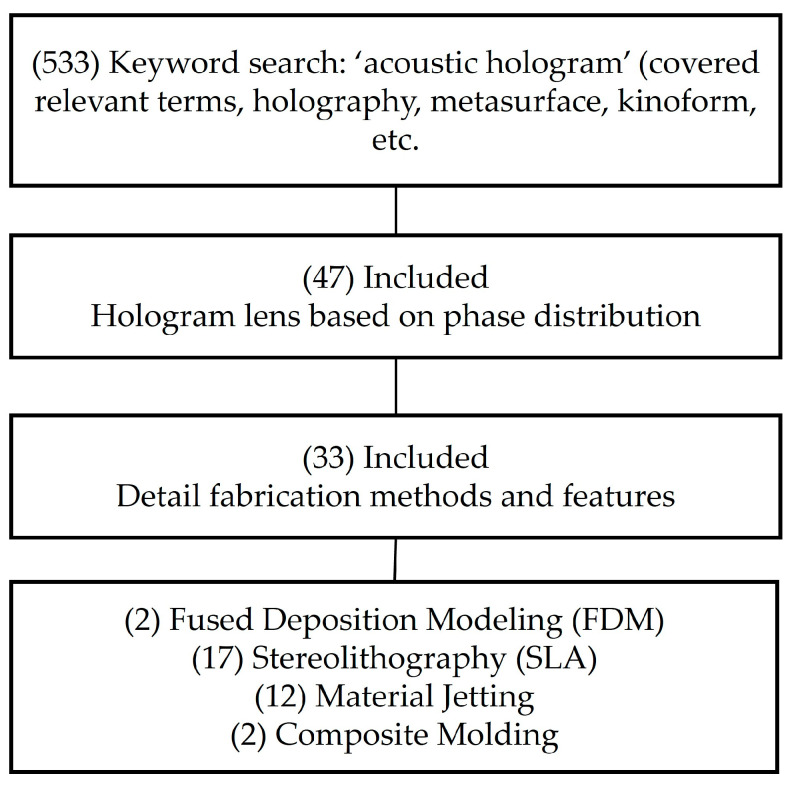
Literature survey flow chart.

We initially searched ScienceDirect, Google Scholar, PubMed, and the public AI tool Perplexity using the keywords “acoustic hologram.” Google Scholar returned the largest number of results (533) including the searched results by other methods, which we then screened to identify papers that included an actual hologram lens as a thin phase-delay map, reducing the set to 47 papers. We excluded studies that did not employ hologram lens components, such as those relying solely on 2D matrix arrays with element-wise voltage/phase control [38], phased-array steering methods [10,39], or thick volumetric hologram structures that involves considerably higher complexity than the source-lens-medium layered propagation consideration [40]. Next, we examined the remaining papers for lens fabrication details, including additive manufacturing approaches, 3D printer models, resin types, and acoustic material properties (e.g., density, sound speed, and attenuation), which filtered out articles that focus on applications and techniques rather than lens microfabrication details [41,42,43]. This filtering yielded 31 papers with sufficient microfabrication details. We then tabulated the key parameters (e.g., operation frequency, printer model, resin properties) and distinctive features of each prototyped lens for its intended application. The survey workflow is summarized in Figure 2.

Overall, the results revealed a dominant trend toward SLA 3D printing and material jetting, both mature and widely adopted techniques capable of high-resolution fabrication (<50 µm). By contrast, only a few studies reported FDM-based methods or composite molding for hologram lens fabrication.

## 3. Acoustic Hologram Microfabrication Methods

### 3.1. Material Jetting 3D Printing

**Figure 3:**
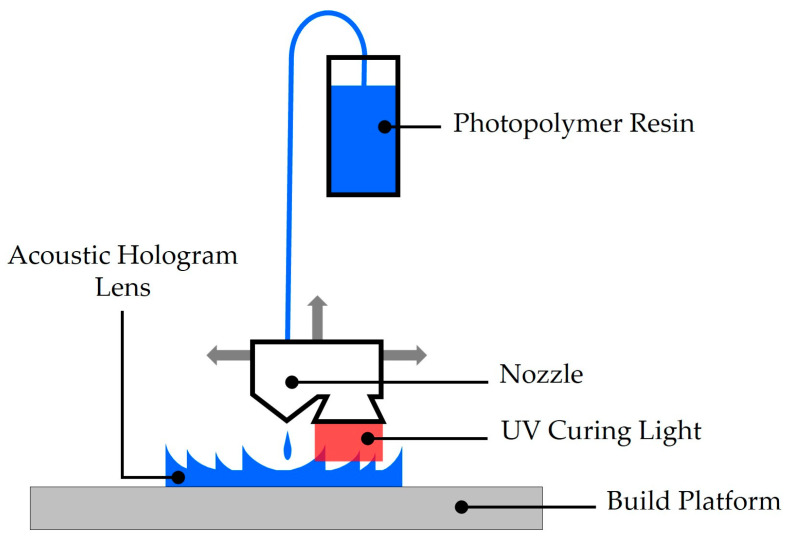
Schematic of acoustic hologram lens fabrication by Material Jetting 3D printing.

Since the first demonstration of acoustic holograms with the terminology “holograms” in 2016, material jetting 3D printing has been widely adopted for fabricating acoustic hologram lenses. Material jetting, often referred to as PolyJet modeling, is an additive manufacturing technique in which liquid photopolymers are jetted as tiny droplets, layer by layer, using a print head similar to that of an inkjet printer [44]. Each deposited layer is immediately cured by ultraviolet (UV) light, allowing precise control over feature resolution. In this process, the photopolymer is stored in an airtight reservoir, heated, and then delivered through a transmission line to the nozzle, where it is deposited as an ultra-thin film on the build platform (Figure 3). The photo-curing step relies on a UV light source of specific wavelength to polymerize monomers and oligomers in the liquid state, solidifying them into the desired structure. This combination of high-precision droplet deposition and rapid photopolymerization enables the fabrication of complex hologram lenses with microscale accuracy [44,45]. The surveyed results on acoustic hologram lens fabrication by material jetting are summarized in Table 1. All the abbreviations are defined the ‘Abbreviations’ at the end of the manuscript.

The key advantages of material jetting, such as multi-material capability, high resolution, and smooth surface finishes, have directly contributed to the high fabrication quality and performance of acoustic hologram lenses [53]. The used frequency range spans from 1.1 MHz to ~4 MHz, and the reported acoustic impedance of the used photopolymer resin ranges from 2.36 MRayl to 2.98 MRayl. With an averaged operating frequency of ~2.4 MHz and the averaged resin wave speed of ~2445 m/s, the reported lateral resolution limit of ~42 µm corresponds to ~0.04λ, which is sufficiently precise for materializing the designed phase profiles. This precision has enabled diffraction-limited focusing and multiplexing of acoustic fields in multiple studies [40].

In addition, the solid, non-porous, and homogeneous structure of lenses fabricated via material jetting has been consistently advantageous. Its multi-material printing capability has also been leveraged for binary and volumetric holograms, where combining waveguide materials with tailored acoustic impedances allows highly accurate wavefront manipulation [40,51].

One common limitation of thin plates produced by material jetting is their susceptibility to out-of-plane bending, warping, or curling, which can arise from thermal gradients during curing. These deformations may be further influenced by build orientation, small thickness, and rapid energy input [54]. Interestingly, in summarized studies (see Table 1), no thermal bending issues were reported for thin acoustic hologram lenses fabricated using this technique, indicating that such challenges may be mitigated under carefully optimized printing conditions.

Certain photopolymers exhibit high acoustic attenuation and suboptimal impedance (determined by density and sound speed), which reduces transmission efficiency through the lens [40,54]. The restricted range of available printable materials poses another challenge, since polymers with higher acoustic impedance would provide better matching between ceramic transducer surfaces and water. In addition, material jetting is generally more expensive than SLA or FDM, and its printed parts are relatively fragile, making them less suitable for pre-stressed applications [1,40,55].

As summarized in Table 1, even the same resin printed on the same machine has been reported to yield slightly different density and wave speed values. Attenuation can vary even more strongly, depending on testing excitation frequency and measurement technique. Such variability introduces uncertainty in acoustic hologram lens design, as accurate material properties are essential for determining the phase-delay map. Consequently, imperfections and performance inconsistencies may arise due to fabrication variability and material property uncertainty [55].

### 3.2. Stereolithography

**Figure 4:**
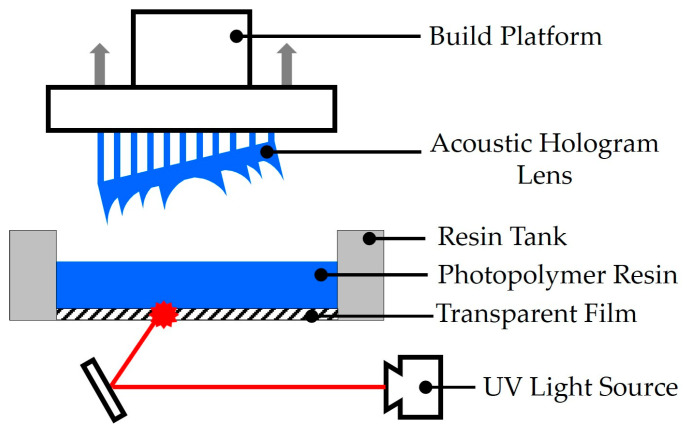
Schematic of acoustic hologram lens fabrication by stereolithography 3D printing.

SLA printing can be classified into top-down and bottom-up approaches, with most modern systems adopting the bottom-up method. All the surveyed acoustic hologram lenses were fabricated using this approach, so our discussion focuses on its mechanism. In bottom-up SLA, a transparent resin tank is filled with liquid photopolymer, and a UV laser selectively cures the resin layer by layer [56]. Printing resolution can be improved by tilting the model during slicing, which reduces the cross-sectional area of each layer (Figure 4). Since the UV laser beam diameter in SLA is typically on the micrometer scale [57,58], this method achieves high spatial resolution and enables the precise fabrication of acoustic hologram lenses.

SLA 3D printing offers key advantages including high resolution, smooth surface finishes, and solid, non-porous, homogeneous structures comparable to those of material jetting [59]. Reported applications span frequencies from 0.5 to ~4.5 MHz, with photopolymer resins exhibiting acoustic impedances between 2.67 and 3.12 MRayl. At an average operating frequency of ~1.4 MHz and an average resin sound speed of ~2503 m/s, the lateral resolution limit of ~50 µm corresponds to ~0.028 λ, providing sufficient precision to accurately reproduce the designed phase profiles.

The main limitations of SLA-fabricated acoustic hologram lenses are the restricted range of printable materials and shrinkage-induced deformation during curing. Achieving efficient ultrasound transmission requires proper acoustic impedance matching between the lens, the ceramic transducer surface, and the coupling medium (e.g., water) [60]. However, SLA resins generally exhibit limited acoustic impedance, and adjusting this property is highly challenging. Material jetting partially overcomes this issue by allowing multi-resin combinations with tailored impedances. Another drawback of SLA is shrinkage deformation, where the thin plate structure of acoustic hologram lenses tends to warp at the edges during photocuring [21,61]. While SLA is typically less expensive than material jetting, it remains costlier than FDM.

As summarized in Table 2, SLA has been widely adopted for acoustic hologram lens fabrication due to its accessibility, relatively high resolution, and overall quality. However, similar to material jetting, studies using even identical resins printed on the same machine reported variations in acoustic properties of acoustic hologram lenses. Such variability undermines consistency and reliability, both of which are crucial for accurate hologram lens fabrication [13,61].

### 3.3. Fused Deposition Modeling

**Figure 5:**
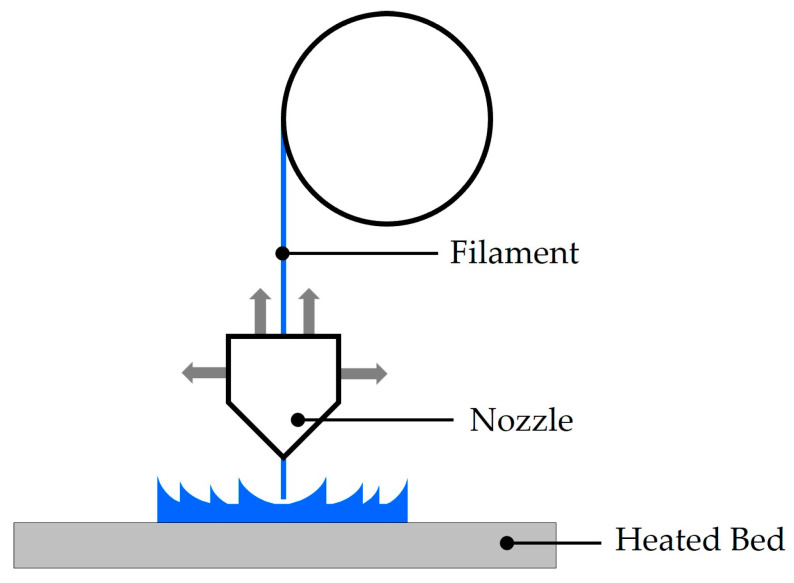
Schematic of acoustic hologram lens fabrication by fused deposition modeling 3D printing.

Fused deposition modeling (FDM) 3D printing builds structures by extruding melted thermoplastic filament through a heated nozzle, which moves in the X–Y plane while the build platform shifts in the Z direction to form successive layers (Figure 5) [75,76]. The process allows adjustment of infill density and the use of various internal infill patterns, such as gyroid, grid, or cubic to tailor structural properties. A key advantage of FDM is its relatively low cost compared to SLA and material jetting.

Despite the distinctive benefits, FDM faces major limitations, including thermal deformation, high porosity, non-homogeneous structures, relatively low resolution, and high sensitivity to process parameters [76,77]. Thermal deformation arises because extruded filaments exit the nozzle at a much higher temperature than the heated bed, cooling rapidly and generating residual stresses that distort the printed part [78]. Furthermore, typical FDM structures are not fully dense but porosities exceeding 50% are common, which excessively reduces acoustic impedance, increases attenuation, and can even allow water leakage when used in underwater experiments. Resolution is also constrained by nozzle diameter, making it lower than SLA or material jetting. Finally, numerous parameters, such as nozzle temperature and bed temperature, must be precisely controlled, adding further complexity.

As summarized in Table 3, only a few studies have employed FDM for acoustic hologram lens fabrication, and the method remains less suitable compared to SLA or material jetting due to its inherent limitations in printing resolution and acoustic properties of thermoplastics [79]. Reported resolutions are the lowest among the three techniques, while the average acoustic impedance of ~2.5 MRayl is slightly lower than that of photopolymers. Combined with the lower wave speed and larger printing resolution, this results in significantly reduced phase profile accuracy relative to the acoustic wavelength. Furthermore, the reported attenuation of 13.72 dB/cm is substantially higher than values observed in SLA and material jetting lenses, further limiting FDM’s effectiveness for acoustic hologram lens fabrication.

### 3.4. Nanoparticle–Epoxy Composite Molding

The 3D printing technology offers cost efficiency and the ability to fabricate complex geometries, which are major advantages over conventional metal machining. However, their utility is limited by the acoustic properties of photopolymers, which are (1) typically not sufficient for optimal wave transmission between hard ceramic transducers and water or air, and by (2) the vulnerability of thin-plate structures, such as acoustic hologram lenses, to thermal deformation during printing. To address these challenges, we previously introduced the nanoparticle–epoxy (NPEC) molding method for acoustic hologram lens fabrication [20,21].

**Figure 6:**
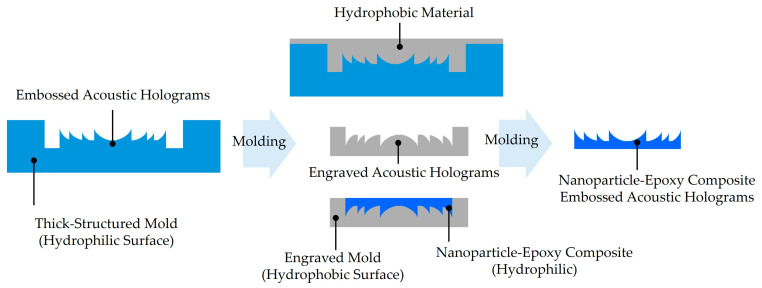
Schematic of acoustic hologram lens fabrication by epoxy composite molding.

NPEC molding approach is designed to utilize the advantages of both cost-efficient, versatile, high-resolution fabrication by SLA printing and acoustic property controllable nanoparticle-epoxy composites. The schematic of NPEC lens fabrication method is depicted in Figure 6.

Briefly, an acoustic hologram surface is designed on the bottom of the bath with a thick back-support, and the bath is 3D printed by SLA printing. After that, the silicone rubber is poured and molded for making a hydrophobic engraved surface for an acoustic hologram mold. Finally, the low-viscosity epoxy and nanoparticle mixture is used through a degassing process to fabricate the NPEC acoustic hologram lens. Despite the one additional molding process included, this subsequent molding approach demonstrated improved lens materialization accuracy with more suitable impedance matched acoustic transmission compared to conventional photopolymer clear resin [11,21]. For the literature survey focusing on this technique, we surveyed using expanded keyword options including ‘epoxy composite, molding, nanoparticle composite’ with acoustic holograms, but the main search results included only our previous studies as summarized in Table 4.

By varying the alumina nanoparticle volume fraction (6.8–22.5%), the acoustic impedance of NPEC lenses was characterized in the range of 2.99–4.64 MRayl, representing a substantial increase over conventional photopolymers while also demonstrating tunability [20,21]. Achieving an impedance of up to 4.64 MRayl without compromising phase-profile accuracy or fabrication speed highlights the overall merit of the NPEC approach for acoustic hologram lens fabrication. The resulting lenses maintained their designed geometry without deformation and exhibited a 1.55-fold higher acoustic impedance compared to standard photopolymers. Experimental demonstrations also showed over 20% enhancement in output acoustic pressure relative to photopolymer-based lenses.

The use of low-viscosity epoxy nanocomposites for acoustic impedance tuning is a well-established method in matching layer and lens fabrication [18,80]. Both heavy-metal and lightweight glass nanoparticles have been explored to achieve a wide impedance range [81]. Extending beyond current results, this strategy enables broader applicability for acoustic hologram lenses, such as in airborne particle trapping or acoustic assembly, which are not feasible with conventional photopolymer lenses [82]. Furthermore, direct nanocomposite printing via SLA offers a promising route to bypass additional molding steps [83]. Incorporating nanoparticles into photocurable resins can enable impedance values exceeding 4 MRayl and introduce additional functionalities, including gradient–impedance structures. However, suspended nanoparticles alter light scattering and absorption during curing, which affects resolution and curing accuracy. Therefore, further optimization of nanocomposite SLA processes is critical to fully realize their potential [83].

## 4. Summary and Discussion

Our survey results clearly indicate that the advancement of acoustic hologram lenses has closely paralleled progress in 3D-printing technologies, particularly in material jetting and SLA [3,17]. By contrast, only a few studies have employed FDM, and while this method shows limited advantages, unexplored opportunities may exist for hybrid lens structures or porosity-assisted lightweight designs. Based on the literature survey, the representative advantages and limitations of additive manufacturing for acoustic hologram lenses can be summarized as follows:

(1) Rapid, one-step fabrication: although post-processing steps such as cleaning and surface polishing are required, current photopolymer-based 3D printing techniques provide sufficiently high resolution (<50 µm) for the typical operating frequency range of 0.5–5 MHz. This resolution is competitive with conventional machining while offering greater accessibility, enabling engineers to fabricate lenses without specialized machining skills [31].
(2) Reliable translation from design to performance: nearly all surveyed studies demonstrated successful realization of predefined multi-focal pressure fields. The accuracy of reconstruction strongly depends on operating frequency and axial alignment, yet both IASA- and ML-assisted design processes combined with 3D printing have proven reliable for custom transducer development, supporting their potential in future applications.
(3) Limited material availability: despite ongoing advances in printable polymers, acoustically optimal material properties remain difficult to achieve with standard 3D printing resins. Workarounds such as NPEC molding illustrate that enhanced material control often comes at the expense of fabrication simplicity and speed [21].
(4) Insufficient thermal stability data: Few studies report detailed thermal reliability of fabricated lenses. Photopolymers typically used in SLA exhibit glass transition temperatures (*T*_g_) in the range of 50–120 °C [84]. Under long-duty, high-voltage operation, lens heating may approach *T*_g_, raising concerns about performance stability in high-power applications. Systematic reliability testing is therefore needed.

Considering these current advantages and limitations, several research opportunities can be identified:

(1) High-power applications: for surgical ultrasound, materials processing, or acoustic manipulation [85,86], high-impedance lens materials (e.g., heavy-particle/epoxy composites, aluminum, stainless steel) may provide improved energy transmission. NPEC molding is one viable approach, while composite SLA or metal 3D printing hold promise for fabricating high-impedance hologram lenses [83,87]. Further analysis of averaged transmission coefficient values across all pixels on the hologram plane for different lens materials could provide useful insights for high-power lens design.
(2) Multi-layered acoustic holograms: inspired by stackable hologram [51], lenses with gradient acoustic impedance across layers could enhance precision and pressure output. Integration with advanced 3D printing techniques may enable further optimization of acoustic transmission.
(3) Dynamic morphing holograms: Additive microfabrication with tissue-like or stimulus-responsive materials could enable lenses capable of reconfigurable focusing. Acoustic properties sensitive to pressure, temperature, magnetic fields, or electric fields could be exploited to create dynamic, adaptive acoustic holograms building upon recent findings on wave speed variation by temperature change [30,88]. This functionality would unlock greater controllability of pressure field distributions at the target volume, expanding the scope of high-precision non-contact excitation applications.

## 5. Conclusions

This review summarized recent advances in microfabrication methods for acoustic hologram lenses, highlighting the strengths and limitations of SLA, material jetting, FDM, and composite molding. SLA and material jetting provide high-resolution, homogeneous structures but remain costly, while FDM is cost-efficient yet limited by porosity and low resolution. Composite molding enables tailored impedance and stable fabrication, though with added complexity. Future directions such as dynamic focusing, metal-based printing, and diverse material integration promise to overcome current trade-offs and further expand applications in biomedical ultrasound, acoustic manipulation, and next-generation multifunctional acoustic devices.

## Figures and Tables

**Table 1:** Survey summary for acoustic hologram lens fabrication results by using material jetting. *ρ*, *v*, and α denote the density, wave speed, and attenuation, respectively. * The used abbreviation list is tabulated in ‘**Abbreviation**’. N/A denotes ‘not applicable.’

Fab. Device	Lens Material	Acoustic Properties	Resolution	Excitation Frequency	Remark *	Ref.
Objet350	Resin VeroBlack	*ρ*: 1190 kg/m^3^ *v*: 2495 m/s	N/A	1.9 MHz 2.5 MHz 3.1 MHz	Multi-frequency kinoform Sim. and Exp. (Hydrophone) Metric: Crosstalk ratio	[2]
Objet350	Resin VeroClear	*ρ*: 1190 kg/m^3^ *v*: 2495 m/s	N/A	<6 MHz	Optoacoustic kinoform using a single optical pulse. Sim. and Exp. (Optical Scanner) Metric: Signal-to-noise ratio	[15]
MJP 2500	Resin Visjet M2R-WT	*ρ*: 1030 kg/m^3^ *v*: 2290 m/s α: 4.6 dB/cm	N/A	1.5 MHz	Simultaneous multi-target blood-brain barrier opening Sim. and Exp. (Hydrophone) Metric: Full-width half-maximum	[46]
Objet 260	Resin VeroClear	N/A	N/A	2 MHz	Combine phased array transducers and static acoustic hologram Sim. and Exp. (Thermochromic sheet) Metric: N/A	[47]
Objet 260	Resin VeroClear	N/A	N/A	2.25 MHz	Particle assembly in 3D space Sim. and Exp. (Trapping microgels) Metric: N/A	[7]
Objet 30	Resin VeroWhite	*ρ*: 1181 kg/m^3^ *v*: 2525 m/s α: 1.65 dB/cm·MHz^−1.1^	N/A	1.66 MHz	Hyperthermia system for multiple Tumor spheroids Sim. and Exp. (Hydrophone/Thermocouple) Metric: Peak-positive-pressures, Temperature, FWHM	[48]
Objet 30	Resin VeroClear	*ρ*: 1191 kg/m^3^ *v*: 2312 m/s α: 3.06 dB/cm	Lateral: 100 µm Axial: 28 µm	1.112 MHz	Focusing self-bending line, volumetric through the skull Sim. and Exp. (Hydrophone) Metric: Overlapping volume, lateral shift of the peak pressure	[4]
Objet350	Resin VeroClear	*ρ*: 1190 kg/m^3^ *v*: 2495 m/s	N/A	2.7 MHz	Modulation phase and amplitude using acoustic hologram Sim. and Exp. (Hydrophone) Metric: amplitude/phase average variation, SNR	[49]
Objet 30	Resin VeroWhite	*ρ*: 1175 kg/m^3^ *v*: 2495 m/s	N/A	2 MHz	Generation acoustic hologram using Deep-learning-based framework Sim. and Exp. (Hydrophone) Metric: CSIM, SSIM, uniformity, PSNR, efficiency	[34]
Objet 260	Resin VeroClear	*v*: 2424 m/s α: 5.5 dB/cm	N/A	2 MHz	Generation acoustic hologram using Deep-learning-based framework Sim. and Exp. (Hydrophone) Metric: CSIM, SSIM, uniformity, PSNR, efficiency	[1]
J750	Resin VeroClear	*ρ*: 1185 kg/m^3^ *v*: 2424 m/s	25 µm	2.3 MHz	Making multifocal beam Sim. and Exp. (Hydrophone) Metric: SNR	[50]
Objet350	Resin TangoBlack and VeroClear	*v_1_*: 1937 m/s *v_2_*: 2495 m/s	N/A	3.0 MHz	Generation of multiple pressure patterns by stacked holograms Sim. and Exp. (Hydrophone) Metric: Peak pressure distribution	[51]
MJP3600	N/A	*ρ*: 1220 kg/m^3^ *v*: 2350 ± 50 m/s	N/A	1.65/1.75 MHz 7.21 MHz 15 MHz	Acoustofluidic holography (AFH) for particle manipulation Sim. and Exp. (Hydrophone and particle patterning test) Metric: MSE	[52]

**Table 2:** Survey summary for acoustic hologram lens fabrication results by using stereolithography. *ρ*, *v*, and α denote the density, wave speed, and attenuation, respectively. * The used abbreviation list is tabulated in ‘**Abbreviation**’. N/A denotes ‘not applicable.’

Fab. Device	Lens Material	Acoustic Properties	Resolution	Excitation Frequency	Remark *	Ref.
Form 2	Resin Clear resin	*ρ*: 1178 kg/m^3^ *v*: 2594 m/s α: 2.92 dB/cm·MHz^−1^	25–100 µm	1 MHz	Holographic thermal mapping in volumes Sim. and Exp. (Hydrophone and IR camera) Metric: PSNR, RMSE, SSIM	[14]
Form 3	Resin Clear resin	*ρ*: 1186 kg/m^3^ *v*: 2599 m/s α: 3.4 dB/cm·MHz^-y^	N/A	500 kHz	Reconstruct 3D acoustic field using 2D measurement Sim. and Exp. (Hydrophone) Metric: Mean Field Difference	[62]
Form 2	Resin Clear resin	*ρ*: 1100 kg/m^3^ *v*: 2424 m/s	50 µm	1 MHz	Multi-focal contactless ultrasonic power transfer system Sim. and Exp. (Hydrophone) Metric: Power enhancement	[13]
nanoArch s140	Resin VeroClear	*ρ*: 1180 kg/m^3^ *v*: 2400 m/s	N/A	1 MHz	Design acoustic hologram with hole Sim. and Exp. (Hydrophone) Metric: Focal length	[63]
Form 2 & 3	Resin Clear resin	*v*: 2430~2650 m/s	125 µm	1.5 MHz 4.5 MHz	Holographic Direct Sound Printing using acoustic hologram Sim. and Exp. (Hydrophone) Metric: PSNR, SSIM	[61]
Form 3	Resin Gray resin	*ρ*: 1178 kg/m^3^ *v*: 2591 m/s α: 2.922 dB/cm at 1 MHz	25 µm	444 kHz	Gradient descent optimization of acoustic holograms Sim. and Exp. (Hydrophone) Metric: Sonication volume, Peak focal pressure	[64]
N/A	Resin	*ρ*: 1178 kg/m^3^ *v*: 2591 m/s	25 µm	1 MHz	Generation of acoustic double vortex in underwater environment Sim. and Exp. (Hydrophone) Metric: N/A	[65]
Form 2	Resin Clear resin	*ρ*: 1171 kg/m^3^ *v*: 2580 m/s α: 1.38 dB/cm	Lateral: 50 µm Axial: 100 µm	0.5 MHz	Transcranial generation of a focused acoustic vortex with hologram lens Sim. and Exp. (Hydrophone) Metric: Normalized amp. and phase	[66]
SLA600	Resin VeroClear	*ρ*: 1300 kg/m^3^ *v*: 2400 m/s	100 µm	3 MHz	Overcoming the limitations of low-fidelity 3D printing with a discrete multi-step phase hologram Sim. and Exp. (Hydrophone) Metric: uniformity, MSE	[67]
Form 3+	Resin White resin	*v*: 2538 m/s	N/A	2 MHz	Encryption of acoustic wave information using acoustic holograms Sim. and Exp. (Hydrophone) Metric: PSNR	[68]
Form 2	Resin Clear resin	*ρ*: 1171 kg/m^3^ *v*: 2580 m/s α: 1.38 dB/cm	50 µm	0.5 MHz	Bilateral focusing through an ex-vivo human skull Sim. amd Exp. (Hydrophone) Metric: Normalized pressure, sonicated volume	[69]
Form 2	Resin Clear resin	*ρ*: 1171 kg/m^3^ *v*: 2580 m/s α: 2.72 dB/cm·MHz^-y^ y = 1.1	25 µm	1 MHz	Design acoustic hologram for ultrasound-induced hyperthermia Sim. and Exp. (IR camera and Hydrophone) Metric: thermal pattern, temperature	[70,71]
N/A	Resin ANY CUBIC Grey resin	*ρ*: 1150 kg/m^3^ *v*: 2352 m/s	XYZ 50 µm 50 µm 70 µm	1.3 MHz	Non-contact rotary ultrasonic motor using acoustic hologram Sim. and Exp. (Motor) Metric: Torque	[72]
N/A	Resin ANY CUBIC Grey resin	*ρ*: 1150 kg/m^3^ *v*: 2352 m/s	N/A	1.3 MHz 1.6 MHz 1.9 MHz	Induce fluid motion using acoustic hologram Sim. and Exp. (Motion of particle) Metric: flow velocity, hydrodynamic force	[73]
Form 2	Resin Clear resin	*ρ*: 1100 kg/m^3^ *v*: 2424 m/s	<20 µm	1 MHz	Cavitation control with acoustic hologram lens Sim. and Exp. (Hydrophone) Metric: MI	[5]
Form 2	Resin Clear resin	*ρ*: 1171 kg/m^3^ *v*: 2580 m/s α: 4.6 dB/cm@1.68 MHz	Lateral: 50 µm Axial: 100 µm	1.68 MHz	Two symmetric foci in vivo for bilateral BBB opening Sim. and Exp. (Hydrophone) Metric: BBB opening volume, PNP, Pressure field distribution, FWHM, Position of focal spot	[60]
Form 3+	Resin UV Sensitive Basic	*ρ*: 1184 kg/m^3^ *v*: 2400 m/s	N/A	2.26 MHz	For high-quality acoustic hologram, apply IASA method applying principles of simulated annealing Sim. and Exp. (Hydrophone and particle patterning) Metric: CSIM, MSE	[74]

**Table 3:** Survey summary for acoustic hologram lens fabrication results by using fused deposition modeling. *ρ*, *v*, and α denote the density, wave speed, and attenuation, respectively. * The used abbreviation list is tabulated in ‘**Abbreviation**’. N/A denotes ‘not applicable.’

Fab. Device	Lens Material	Acoustic Properties	Resolution	Excitation Frequency	Remark *	Ref.
Ultimaker 3 Extended	N/A	*ρ*: 1127 kg/m^3^ *v*: 1818 m/s α: 13.72 dB/cm	100 µm	1.11 MHz	Focusing multiple focal points through a skull Sim. and Exp. (Hydrophone) Metric: N/A	[4]
N/A	N/A	*ρ*: 1184 kg/m^3^ *v*: 2495 m/s α: 2.66 Np/cm	Lateral: 750 µm Vertical: 250 µm	2 MHz	Generation single and dual focus transcranial ultrasound focusing Sim. and Exp. (Hydrophone) Metric: Target registration errors, FWHM (in target/out target ratio)	[77]

**Table 4:** Survey summary for acoustic hologram lens fabrication results by using nanoparticle–epoxy composite molding. *ρ*, *v*, and α denote the density, wave speed, and attenuation, respectively. * The used abbreviation list is tabulated in ‘**Abbreviation**’.

Fab. Device	Lens Material	Acoustic Properties	Resolution	Excitation Frequency	Remark *	Ref.
Form 3 Silicone rubber mold	Alumina-Epoxy composite	*ρ*: 1680 kg/m^3^ *v*: 2763 m/s α: 0.42 dB/mm	<50 µm	1.5 MHz	Lens thickness profile comparison: clear resin vs. NPEC Sim. and Exp. (Hydrophone) Metric: Cross-correlation, SSIM	[20,21]
Form 3 Silicone rubber mold	Alumina-Epoxy composite	*ρ*: 1127 kg/m^3^ *v*: 1818 m/s	<50 µm	0.8 MHz	Multi-focal 4 × 4 well pate sonicator prototype Sim. and Exp. (Hydrophone) Metric: Cross-correlation	[11]

## Data Availability

Not applicable.

## Abbreviations

The following abbreviations are used in this manuscript:

SNR	Signal to noise ratio
PSNR	Peak signal to noise ratio
FWHM	Full width half maximum
MSE	Mean square error
RMSE	Root mean square error
SSIM	Structural similarity index
CSIM	Color similarity index
PNP	Peak negative pressure
MI	Mechanical index
